# Molecular analysis of cyclic α-maltosyl-(1→6)-maltose binding protein in the bacterial metabolic pathway

**DOI:** 10.1371/journal.pone.0241912

**Published:** 2020-11-19

**Authors:** Masaki Kohno, Takatoshi Arakawa, Naoki Sunagawa, Tetsuya Mori, Kiyohiko Igarashi, Tomoyuki Nishimoto, Shinya Fushinobu

**Affiliations:** 1 Department of Biotechnology, The University of Tokyo, Tokyo, Japan; 2 R&D Division, HAYASHIBARA CO., LTD., Okayama, Japan; 3 Collaborative Research Institute for Innovative Microbiology, The University of Tokyo, Tokyo, Japan; 4 Department of Biomaterial Sciences, The University of Tokyo, Tokyo, Japan; 5 VTT Technical Research Centre of Finland Ltd., Espoo, Finland; Universidade Nova de Lisboa Instituto de Tecnologia Quimica e Biologica, PORTUGAL

## Abstract

Cyclic α-maltosyl-(1→6)-maltose (CMM) is a cyclic glucotetrasaccharide with alternating α-1,4 and α-1,6 linkages. Here, we report functional and structural analyses on CMM-binding protein (CMMBP), which is a substrate-binding protein (SBP) of an ABC importer system of the bacteria *Arthrobacter globiformis*. Isothermal titration calorimetry analysis revealed that CMMBP specifically bound to CMM with a *K*_d_ value of 9.6 nM. The crystal structure of CMMBP was determined at a resolution of 1.47 Å, and a panose molecule was bound in a cleft between two domains. To delineate its structural features, the crystal structure of CMMBP was compared with other SBPs specific for carbohydrates, such as cyclic α-nigerosyl-(1→6)-nigerose and cyclodextrins. These results indicate that *A*. *globiformis* has a unique metabolic pathway specialized for CMM.

## Introduction

ABC transporters constitute a membrane protein family that mediates diverse ATP-driven transport [1]. They basically consist of a pair of cytoplasmic domains, called ATP-binding cassettes (ABC) or nucleotide-binding domains (NBD), and transmembrane domains (TMDs) in the cell membrane [2]. ABC importers facilitate efficient intakes of substrates across the cell membrane, and bacterial importers usually work in conjunction with respective substrate-binding protein (SBP) that binds the substrate [3]. As a general transport mechanism of bacterial ABC importers, SBP specifically captures a substrate and delivers it to TMDs, and ATP hydrolysis by NBDs drives conformational changes of TMDs that translocate the substrate into the cytoplasm. To date, various SBPs that bind various carbohydrates, amino acids, ions, and other compounds have been identified, and they share similar three-dimensional structures despite their low amino acid sequence homology [4]. SBPs have a highly conserved structural fold that consists of two α/β domains with a central β-sheet flanked by α-helices. The two α/β domains are connected by a hinge region. On substrate binding, conformations of these domains largely change to switch the state from open to closed, with the ‘‘Venus Fly-trap” mechanism [5]. SBPs in gram-positive bacteria, which include *Arthrobacter* species, are anchored to lipids in the cell membrane [6].

**Fig 1:**
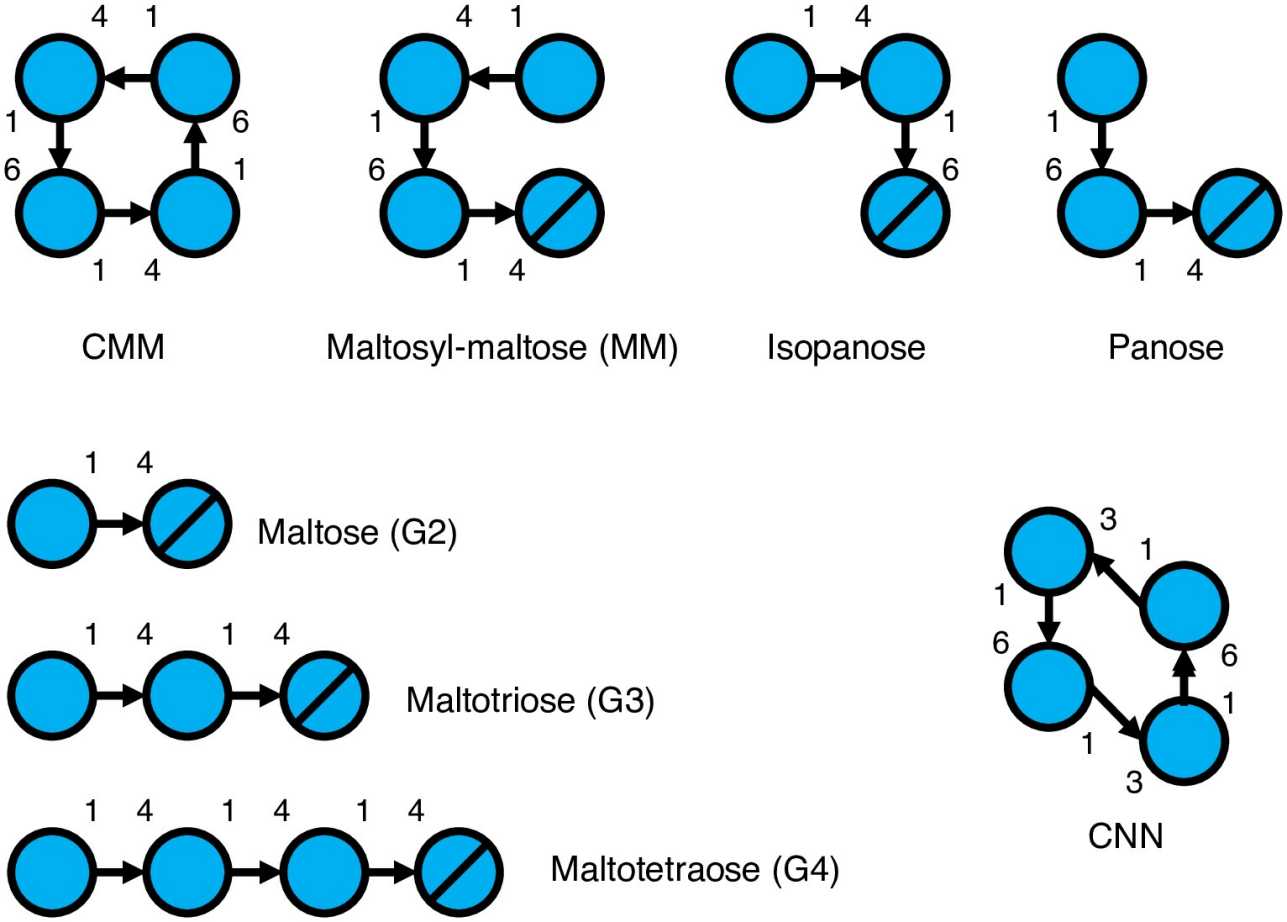
Schematic models of the oligosaccharides appearing in this study. The ligands (oligosaccharides) used for native PAGE (Fig 2) and cyclic α-nigerosyl-(1→6)-nigerose (CNN). Circles and slashed circles indicate glucose and reducing end glucose, respectively.

Cyclodextrins and other cyclic oligosaccharides have ability to increase solubility and stability of various guest molecules, to mask odors and change their physical properties [7]. Therefore, cyclic oligosaccharides potentially have applications in various fields, such as food, cosmetics, pharmaceutical, chemical, textile, and agricultural industries. Cyclic α-maltosyl-(1→6)-maltose [CMM, cyclo-{→6}-α-d-Glc*p*-(1→4)-α-d-Glc*p*-(1→6)-α-d-Glc*p*-(1→4)-α-d-Glc*p*-(1→)] is composed of two maltose units with two α-1,6 linkages (Fig 1). Along with cyclic α-nigerosyl-(1→6)-nigerose (CNN or cycloalternan, see Fig 1) [8, 9], CMM is one of the smallest cyclic glucosaccharides (tetramers) that can be synthesized using enzymes. We have previously identified a novel starch utilization pathway via CMM in *Arthrobacter globiformis* M6 [10]. The gene cluster of the CMM metabolic pathway consisted of seven open reading frames (*cmmA*–*G*) [11], and three enzymes belonging to glycoside hydrolase family 13 are involved in the formation and degradation of CMM. An extracellular enzyme, 6-α-maltosyltransferase (CmmA), produces CMM by inter- and intramolecular α-1,6-transglucosylation [10], and two intracellular enzymes, CMM hydrolase (CmmF) [12] and α-glucosidase (CmmB), synergistically degrade CMM to glucose [13]. We previously reported the mechanistic details of the hydrolysis of CMM by CMM hydrolase with its crystal structures [14]. Gene products encoded by *cmmC*, *cmmD*, and *cmmE* were suggested to form an ABC transporter system of an SBP (CmmC) and TMDs (CmmD and CmmE) [11]. Although this transporter was presumed to import CMM across the cell membrane in CMM metabolism in *A*. *globiformis* M6, its molecular function has not yet been studied. In this study, we performed functional and structural analyses of CmmC by gel shift assay, isothermal titration calorimetry (ITC), and X-ray crystallography. Because our study revealed that the protein specifically binds to CMM, CmmC was termed CMM-binding protein (CMMBP).

## Materials and methods

### Protein preparation

The CMMBP-encoding gene (*cmmC*) was amplified from pBlue-T1 [11] by PCR to express it as an N-terminally His-tagged (6×His) protein and inserted between the NdeI and BamHI sites of the pET-28b (+) vector (Novagen, Madison, WI, USA). *Escherichia coli* Rosetta2 (DE3) (Novagen) transformed by the expression plasmid was cultured in lysogeny broth (LB) medium containing antibiotics (50 mg/L kanamycin and 34 mg/L chloramphenicol) at 37°C until O. D_600 nm_ = 0.6. To induce protein expression of the transformant, 0.1 mM (final concentration) isopropyl 1-thio-β-D-galactopyranoside (FUJIFILM Wako Pure Chemical Co., Osaka, Japan) was added to the medium. The medium was further cultured at 15°C for 24 h. The cells were harvested by centrifugation at 10,000 *g* for 15 min at 4°C and suspended in 50 mM Tris-HCl (pH 8.0) and 500 mM NaCl. To obtain cell-free extracts, the suspended solution was sonicated and centrifuged at 38,000 *g* for 45 min at 4°C. The supernatant was filtered using a 0.45-μm filter and further purified by nickel affinity column chromatography using cOmplete His-Tag Purification Resin (Sigma-Aldrich Co., St. Louis, MO, USA) with two elution steps of 20 and 500 mM imidazole in 50 mM Tris-HCl (pH 8.0). After buffer exchange using an ultrafiltration centrifugal membrane unit (Vivaspin Turbo, 10 kDa molecular weight cutoff; Sartorius Stedim Biotech, Göttingen, Germany), the protein was further purified using a HiTrap Q HP column with a linear gradient from 0 to 1 M NaCl in 20 mM Tris-HCl (pH 8.0). For crystallization, the protein was further purified using a HiLoad 16/60 Superdex 200 pg (GE Healthcare, Buckinghamshire, England). For gel filtration column chromatography, the protein sample was concentrated using an ultrafiltration centrifugal membrane unit (Vivaspin Turbo, 10 kDa molecular weight cutoff; Sartorius Stedim Biotech). The loading buffer was 20 mM Tris-HCl (pH 8.0) and 150 mM NaCl. The protein was eluted by loading buffer supplemented with 1% (w/v) TETRUP^®^ (HAYASHIBARA CO., LTD., Japan). The content of TETRUP^®^ is as follows: 2% glucose, 6.9% maltose, 10.7% maltotriose, 53% maltotetraose, and 28% dextrin (longer maltooligosaccharides). Although MM or panose was not detected in TETRUP^®^ in our detailed analysis, we presumed that such α-1,6-linked oligosaccharides were contained in the gel filtration column. This is because we previously used the same column for purification of 6-α-maltosyltransferase [10], which can produce MM from maltooligosaccharides, and TETRUP^®^ was also used for the previous elution step. The protein concentration was determined by a NanoDrop ND-1000 spectrophotometer (Thermo Fisher Scientific, Waltham, WA, USA) using the extinction coefficient ε_280 nm_ = 89,380 M^-1^cm^-1^, which was estimated from the amino acid sequence of the His-tagged recombinant protein.

### Gel shift assay and ITC analysis

Pinedex^®^ #100 (partial hydrolysate of starch used for gel shift assay) was purchased from Matsutani Chemical Industry Co., Ltd. (Hyogo, Japan). Dextran and pullulan were purchased from FUJIFILM Wako Pure Chemical Co. For native PAGE, a 10% (w/v) lower gel with or without 0.5% (w/v) polysaccharides was used. The ligand solution contained 100 mM CMM, MM, isopanose, panose, G2, G3, or G4. Samples loaded on the PAGE (10 μL) contained 2.5 μL native-PAGE sample buffer (4×), 1 μL CMMBP (2.5 mg/mL in 20 mM Na-acetate, pH 6.0), and 6.5 μL ligand solution (or H_2_O). The bovine serum albumin (BSA) standard sample (9 μL) contained 2.5 μL native-PAGE sample buffer (4×), 2.5 μL BSA (1 mg/ml in H_2_O), and 4.0 μL H_2_O. The 4× native-PAGE sample buffer contained 250 mM Tris-HCl (pH 6.8), 40% glycerol and 0.1% bromophenol blue.

For ITC measurements, a purified CMMBP protein sample was extensively dialyzed against 20 mM Tris-HCl (pH 8.0), and the ligands were dissolved in the same buffer to minimize the heat of dilution. ITC measurements were performed at 25°C using Micro-Cal VP-ITC (Malvern Instruments Ltd., Malvern, Worcestershire, UK). The protein solution (10.6 and 82.3 μM for measurements of CMM and MM titration, respectively) was stirred at 300 rpm in a 1.44-ml cell and titrated with 10 μL of a ligand solution (0.15 and 2.0 mM for CMM and MM, respectively) 25 times at intervals of 420 s. Calorimetric data were analyzed using MicroCal Origin 7.0 (Light Stone, Tokyo, Japan). Thermodynamic parameters, such as association constants (*K*_a_), binding enthalpy (*ΔH*), and the number of binding sites (*n*), were determined by fitting data into a one-site binding model. Changes in Gibbs binding free energy (*ΔG*°), dissociation constants (*K*_d_), and binding entropy changes (*ΔS*°) were calculated from the equations: *ΔG*° = –*RT*ln*K*_a_ = *RT*ln*K*_d_ and *ΔG*° = *ΔH*–*TΔS*°, where *R* and *T* are the gas constant and absolute temperature (298.15 K), respectively. We assumed that *ΔH* values determined from ITC are equal to the standard enthalpy change (*ΔH*°).

### Crystallography

The protein used for crystallization was purified using gel filtration chromatography, eluted with a buffer containing maltotetraose-rich syrup (see above). Crystallization was performed by the sitting drop vapor diffusion method at 20°C by mixing 0.5 μL of a protein solution (15 mg/mL CMMBP in 10 mM Tris-HCl pH 8.0) and an equal volume of a reservoir solution containing 0.1 M Tris-HCl (pH 8.5) and 2.0 M ammonium sulfate. The crystals were cryoprotected in the reservoir solution supplemented with 20% (v/v) PEG400. The X-ray diffraction data were collected under the 100 K cryogenic nitrogen stream at AR-NW12A beamline of the Photon Factory in the High Energy Accelerator Research Organization (KEK, Tsukuba, Japan). The wavelength of X-ray was set at 1.0000 Å. The diffraction images were processed with XDS [15]. Molecular replacement was performed with MOLREP [16]. The model was further built manually with COOT [17] and refined with REFMAC5 [18]. A polder map [19] was created using PHENIX. Molecular graphic images were prepared using PyMOL (Schrödinger LLC, New York, NY, USA). Sequence conservation mapping was performed using the ConSurf server [20].

## Results and discussion

### Functional analysis of CMMBP

**Fig 2:**
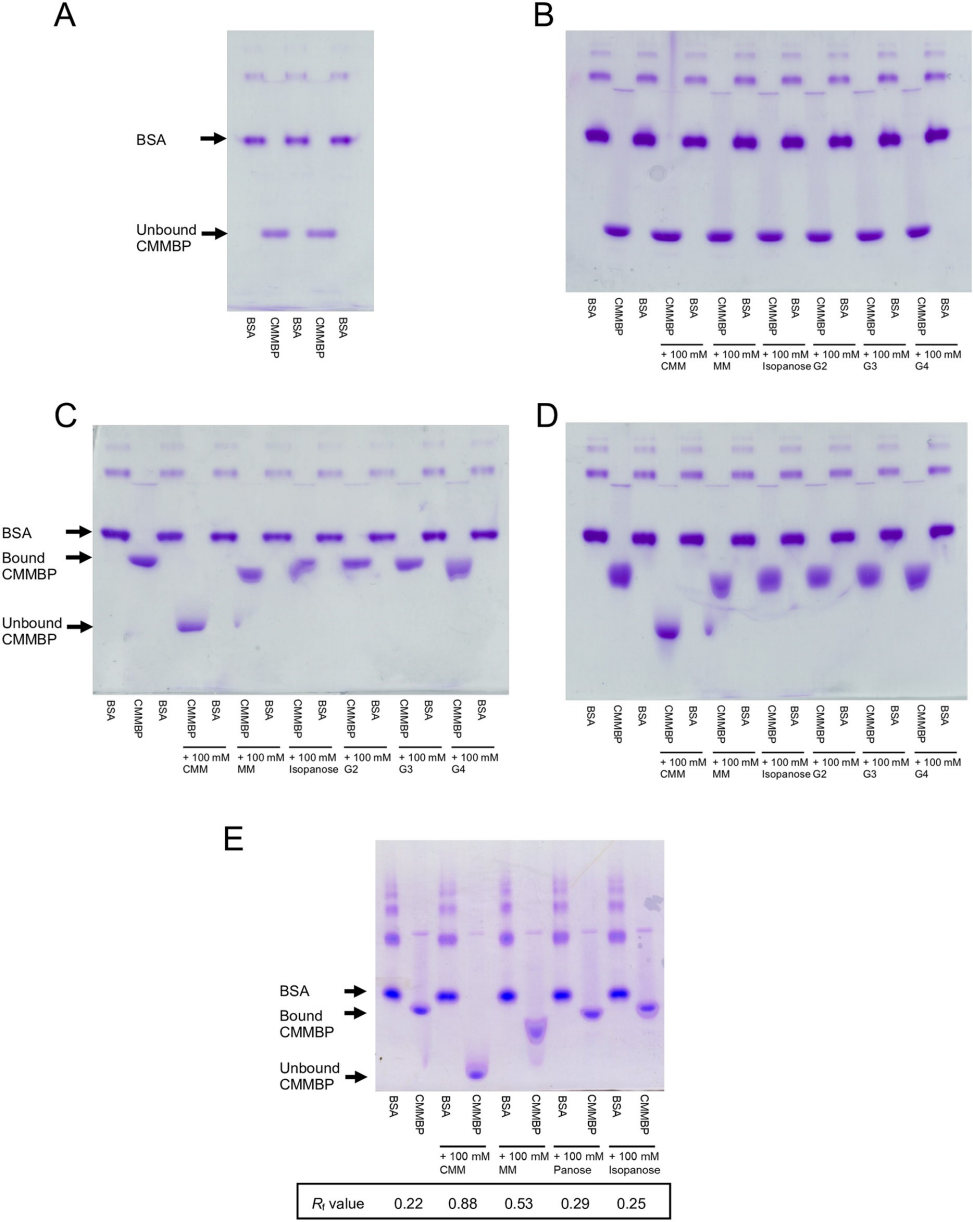
Gel shift assays. Native-PAGE without polysaccharides (A) and in the presence of Pinedex #100 (B), dextran (C and E), and pullulan (D) are shown. The concentrations of the polysaccharides were 0.5% (w/v). For (B–E), samples containing 100 mM oligosaccharides were also used. BSA was used as a control protein. (E) The *R*_f_ values were calculated as fraction of the band position of CMMBP from BSA (offset: 0.00) to the front (1.00).

The substrate-binding ability of CMMBP was preliminarily examined by gel shift assays using polysaccharides and probable ligands (oligosaccharides). The mobility of CMMBP (Fig 2A) was not affected in the presence of Pinedex #100 (partial hydrolysate of starch that is mainly linked by α-1,4 linkages, 1.3%-hydrolysis, Fig 2B), regardless of whether any oligosaccharides were added. On the other hand, in the presence of dextran (>90% α-1,6 linkages) and pullulan (polymer of maltotriose connected with α-1,6 linkage), CMMBP exhibited reduced mobility (Fig 2C and 2D), suggesting that CMMBP interacts with those polysaccharides. In the competition assay with the oligosaccharide ligands, CMMBP combined with CMM showed the same mobility as in the polysaccharide-free gel. This result indicates that CMMBP strongly binds to CMM, and thus interaction with the polysaccharides was canceled. The addition of α-maltosyl-(1→6)-maltose (MM) slightly changed the mobility, suggesting lower affinity of CMMBP to MM. There was no significant effect with other oligosaccharide ligands, namely, isopanose, maltose (G2), maltotriose (G3), and maltotetraose (G4). Then, we performed a gel shift assay in the presence of dextran to quantitively measure the binding competition effect of the oligosaccharides on CMMBP (Fig 2E). In addition to CMM, MM, and isopanose, panose was included in this assay. The relative to front (*R*_f_) value indicated that the binding competition effect was CMM (0.88) > MM (0.53) > panose (0.29) and isopanose (0.25).

**Fig 3:**
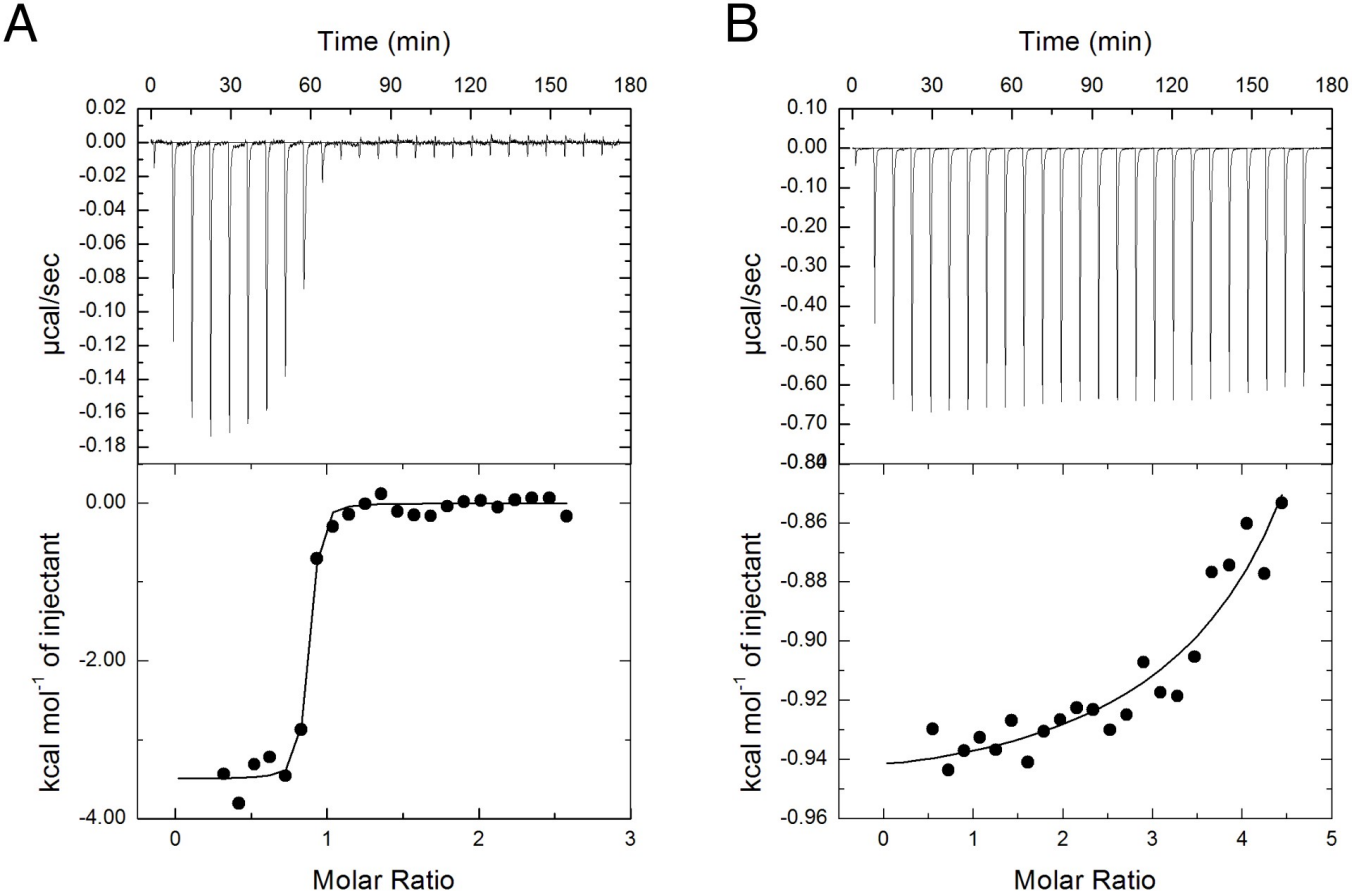
ITC of CMMBP binding for CMM (A) and MM (B). Titration thermograms (top) and binding isotherms (bottom) are shown. Assay conditions are described in the Materials and Methods.

Subsequently, we measured the binding affinity and thermodynamic parameters for CMM and MM using ITC (Fig 3 and Table 1). The binding of CMM was both enthalpy- and entropy-driven with an association constant (*K*_a_) of 1.04 × 10^8^ M^–1^, which corresponds to a *K*_d_ value of 9.6 nM. Heat pulses were also observed with titration of MM, but the isotherm curve was not sigmoidal. Although the parameters were not accurately determined due to the weak affinity and the low *c*-window value, the *K*_a_ value for MM was estimated to be ~500-fold lower than that for CMM.

**Table 1:** Affinity and thermodynamic parameters of ligand binding to CMMBP at 25°C estimated by ITC.

Ligand	*K*_a_ (×10^8^ M^–1^)	*ΔG*° (kcal mol^–1^)	*ΔH* (kcal mol^–1^)	*TΔS*° (kcal mol^–1^)	*n*	*c*
CMM	1.04 ± 0.46	−10.93	−3.49 ± 0.068	−7.44	0.833 ± 0.007	352
MM ^a^	(0.0022 ± 0.0014)	(−7.29)	(−0.95 ± 0.008)	(−6.34)	(6.27 ± 0.876)	1.03

The *c*-window was calculated as follows: *c* = *nK*_a_[MM].

^a^ Values are not reliable due to the weak affinity and the low *c* value.

### Crystal structure of CMMBP

**Fig 4:**
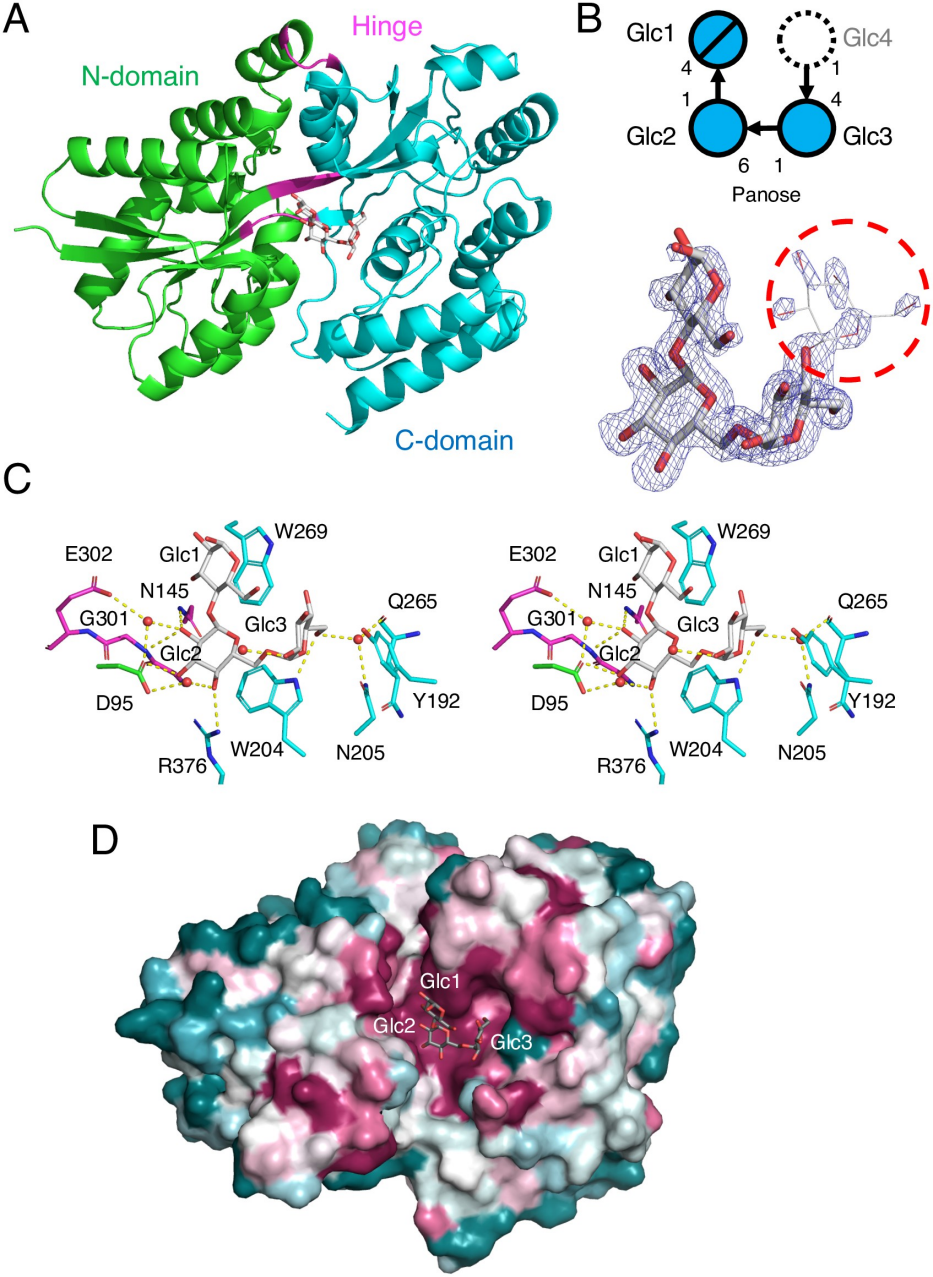
Crystal structure of CMMBP. (A) Overall structure. The N-domain, C-domain, and hinge regions are colored in green, cyan, and magenta, respectively. Panose is shown as gray sticks. (B) Schematic presentation (top) and polder map (3.5σ) of the ligand (bottom). If the glucose unit at the nonreducing end (Glc4, indicated by a dashed-line circle) is included, the molecule becomes α-maltosyl-(1→6)-maltose (MM). (C) Stereographic view of the ligand binding site. Protein residues are colored as in panel (A). (D) Sequence conservation mapping on the molecular surface. Amino acid sequence conservation among CMMBP homologs (identity > 35%) is colored with red (high), white (middle), and blue (low).

Although our crystallization trials of CMMBP in the presence of CMM failed, crystals were grown without any substrates in the mother liquors. The crystal structure of CMMBP was solved by molecular replacement using the structure of an uncharacterized SBP from *Thermotoga lettingae* (PDB ID: 5CI5) as a template. The CMMBP crystal contained one molecule in an asymmetric unit, and the refined structure was determined at a resolution of 1.47 Å (Table 2). CMMBP adopts a typical “SBP fold” consisting of two α/β domains, designated as the N- and C-domains (Fig 4A). The N-domain (residues 31–143 and 303–358) consists of a five-stranded β-sheet flanked by nine α-helices, and the C-domain (147–299 and 362–421) consists of a three-stranded β-sheet flanked by nine α-helices. The two domains are connected by three hinge regions (144–146, 300–302, and 359–361), and a long β-strand penetrates both domains. Although we did not add any ligands at the crystallization step, an electron density of three or four glucose residues was observed in the cleft between the two domains (Fig 4B). The tetrasaccharide appears to be connected by the central α-1,6 linkage and two flanking α-1,4 linkages, suggesting that this compound is a MM. Because the electron density of the glucose unit at the nonreducing end (Glc4) was ambiguous, we modeled a trisaccharide (Glc1–3), which corresponds to a panose, in the crystal structure (thick sticks in Fig 4B). There was no significant interaction at the putative binding site for Glc4, probably because the structure is in an open conformation (discussed below). We presume that the gel filtration column contained a small amount of MM and panose (see Materials and methods).

**Table 2:** Data collection and refinement statistics of the crystallography.

Data set	CMMBP
Data collection ^a^	
Space group	*P*6_1_22
Unit cell (Å/°)	*a* = *b* = 92.08, *c* = 161.58
Resolution (Å)	46.04–1.47 (1.50–1.47)
Total reflections	1,350,030 (32,216)
Unique reflections	68,976 (3,231)
Completeness (%)	99.5 (95.8)
Multiplicity	19.6 (10.0)
Mean *I/σ*(*I*) ^b^	15.7 (1.2)
*R*_merge_	0.093 (1.794)
CC_1/2_	0.981 (0.930)
Refinement	
Resolution (Å)	44.67–1.47
No. of reflections	65,458
*R*_work_/*R*_free_	0.184/0.206
Number of atoms	3,434
RMSD from ideal values	
Bond lengths (Å)	0.013
Bond angles (°)	1.778
Ramachandran plot (%)	
Favored/allowed/outlier	99.0/1.0/0.0
PDB ID	7BVT

^a^ Values in parentheses are for the highest resolution shell.

Fig 4C shows the interactions between CMMBP and panose. Glc1 forms a stacking interaction with Trp269, and no hydrogen bond interaction was observed. Glc2 forms a stacking interaction with Trp204 and several hydrogen bonds. Direct hydrogen bonds are formed with the side chains of Asp95, Asn145, and Arg376 and the main chain of Gly301. Glc3 forms a relatively distant stacking interaction with Tyr192 and a direct hydrogen bond with Trp204. The Glc2 and Glc3 moieties form water-mediated hydrogen bonds with Asn205, Gln265, and Glu302. The residues involved in the interactions are mainly from the C-domain (cyan in Fig 4C), and the hinge-region (magenta) makes several polar interactions with Glc2. The degree of amino acid sequence conservation among homologous proteins on the database was mapped on the molecular surface of CMMBP (Fig 4D) This result illustrated that the residues forming interactions with the ligand in the cleft are highly conserved. The thermodynamic parameters in Table 1 shows that the binding process of CMMBP is dominantly driven by the favorable entropy change. This feature is consistent with the ligand interactions of CMMBP, in which three stacking (hydrophobic) interactions can influence on the affinity (Fig 4C). The entropy-driven thermodynamics of maltose (or maltodextrin)-binding protein from *E*. *coli*, which has a large hydrophobic cleft, have been explained by the release of a large number of ordered water molecules upon substrate binding [21]. The hydrogen bonds mediating ligand binding may contribute to the smaller but favorable enthalpy change of CMMBP.

### Comparison with other SBPs

**Fig 5:**
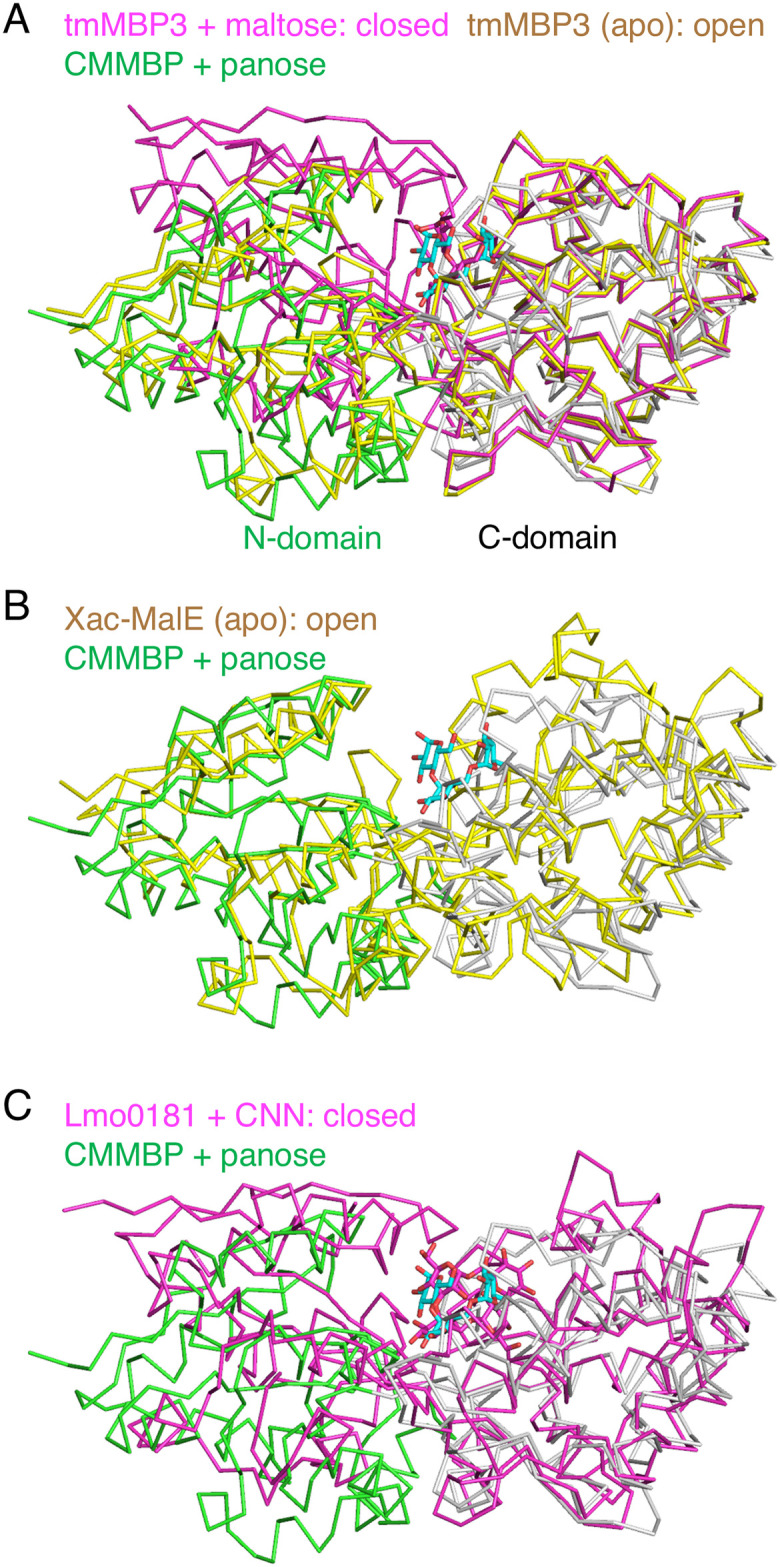
Structural comparison of CMMBP with tmMBP3 (A), Xac-MalE (B), and Lmo0181 (C). Cα traces of CMMBP (green for the N-domain and hinge and gray for the C-domain), tmMBP3 plus maltose (PDB ID: 6DTQ, magenta), unliganded state of tmMBP3 (6DTR, yellow), Xac-MalE (PDB ID: 3UOR, yellow) and Lmo0181 plus CNN (5F7V, magenta) are shown. The structures were superimposed with the C-domain.

A database search using the DALI server [22] revealed that CMMBP shows high structural similarity to SBPs that bind maltooligosaccharides (or other α-glucosides) and other sugar compounds (Table 3). The best match was a maltose binding protein 3 from *Thermotoga maritima* (tmMBP3) [23] and a putative maltose/trehalose-binding protein from *Xanthomonas citri* (Xac-MalE) [24]. We noticed that the binding protein for CNN from *Listeria monocytogenes* (Lmo0181) also exhibited structural similarity to CMMBP [25]. CNN is the other type of cyclic glucotetrasaccharide linked by alternating α-1,3 and 1,6 linkages (Fig 1). Fig 5A shows superimposition of the CMMBP structure with the open (unliganded) and closed (maltose complex) structures of tmMBP3 by alignment of Cα atoms in the C-domain. As evident from the position of the N-domains, the CMMBP structure adopts an open conformation. Superimpositions with Xac-MalE (open state without ligand) and Lmo0181 (closed state complexed with CNN) also suggested that CMMBP has an open conformation (Fig 5B and 5C).

**Table 3:** Results of the structural similarity search using the Dali server.

PDB ID^a^	Z score	RMSD (Å)	LALI^b^	Identity (%)	Protein name	Organism	Binding specificity	Reference
6DTR (A)	43.7	2.5	376	23	tmMBP3	*Thermotoga maritima*	Maltotetraose	[23]
3UOR (A)	42.0	2.5	370	21	Xac-MalE	*Xanthomonas citri*	Unknown	[24]
4MFI (A)	41.3	2.7	378	20	UgpB	*Escherichia coli*	*sn*-Glycerol-3-phosphate	[26]
4RJZ (A)	41.3	2.6	365	25	ATU4361	*Agrobacterium fabrum*	Maltooligosaccharides	TBP
6JAL (A)	41.1	2.5	374	21	αGlyBP	*Thermus thermophilus*	α-glucosides	[27]
5CI5 (B)	41.0	3.8	369	27	Tlet_1705	*Thermotoga lettingae*	α-D-Tagatose	TBP
4QRZ (A)	41.0	3.6	367	25	ATU4361	*Agrobacterium fabrum*	Maltooligosaccharides	TBP
5YSB (A)	41.0	2.6	365	21	SO-BP	*Listeria innocua*	β-1,2-glucooligosaccharide	[28]
3K01 (A)	40.9	2.4	369	23	GacH	*Streptomyces glaucescens*	Acarbose	[29]
1EU8 (A)	39.8	3.7	373	23	TMBP	*Thermococcus litoralis*	Trehalose/maltose	[30]
5F7V (A)	37.9	3.6	366	25	Lmo0181	*Listeria monocytogenes*	CNN	[25]
4GQO (B)	37.7	2.7	379	20	Lmo0859	*Listeria monocytogenes*	Unknown	TBP
2ZYO (A)	37.0	2.5	365	16	TvuCMBP	*Thermoactinomyces vulgaris*	Cyclo/maltodextrin	[31]

TBP, to be published. Proteins appearing in the main text are underlined.

^a^Chain ID is shown in parentheses.

^b^Number of aligned residues.

**Fig 6:**
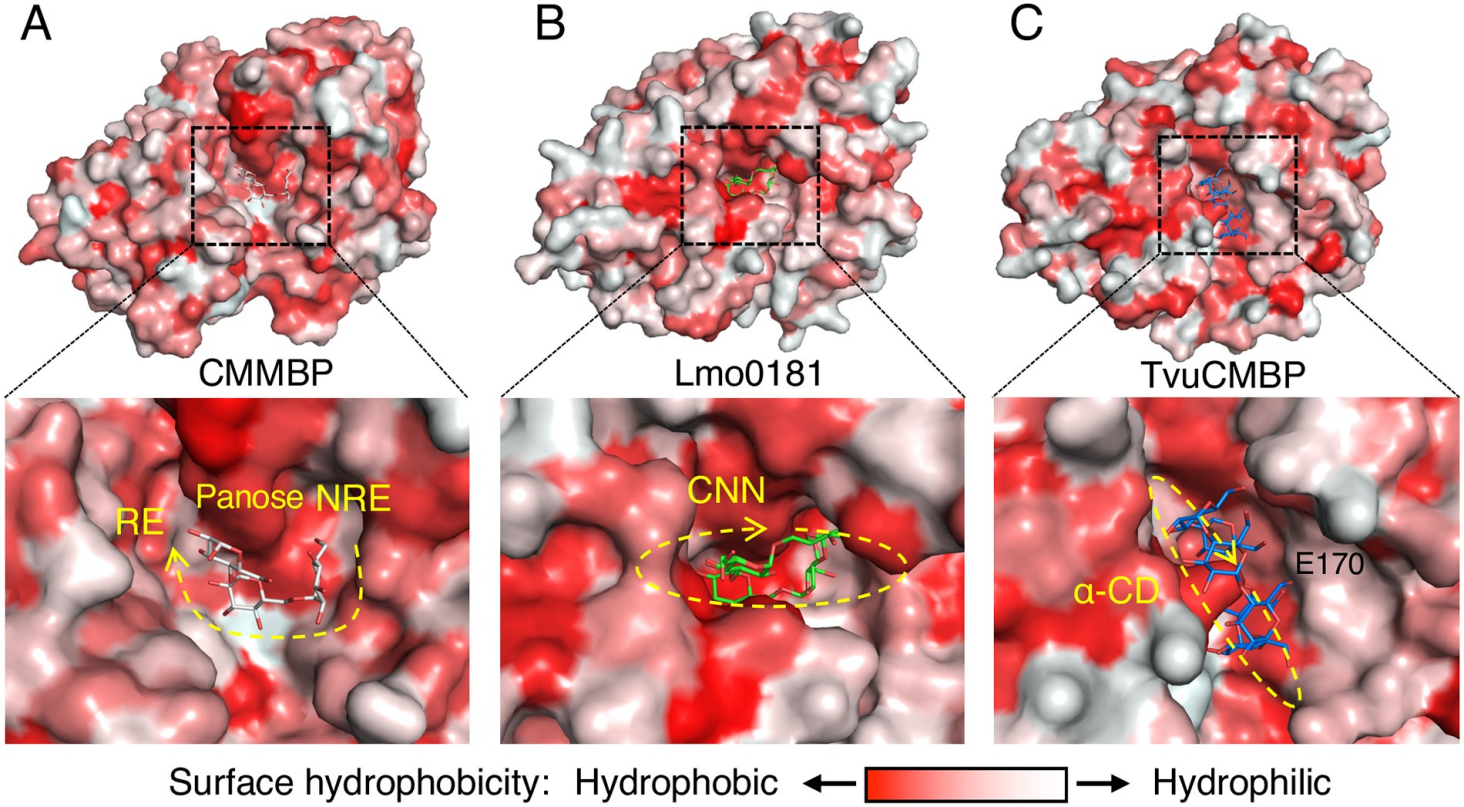
Comparison of the substrate binding cleft with other SBPs. Surface representation of CMMBP (A), Lmo0181 (B, 5F7V), and TvuCMBP (C, 2ZYM) are shown. The surfaces are colored by hydrophobicity. Yellow dashed lines indicate the direction of the ligands from the nonreducing end (NRE) side to the reducing-end (RE) side. α-CD, α-cyclodextrin (cyclic glucohexasaccharide).

The substrate-binding cleft of CMMBP was compared with other SBPs that bind cyclic glucooligosaccharides (Fig 6). Cyclo/maltodextrin-binding protein from *Thermoactinomyces vulgaris* (TvuCMBP) [31] was included in this comparison because it showed a structural similarity with CMMBP comparable to Lmo0181 (Table 3). Surface representations of CMMBP, Lmo0181, and TvuCMBP illustrated that they generally have a large and hydrophobic cleft. The binding cleft of TvuCMBP is narrow and long, and the binding orientation of cyclodextrin is perpendicular to the major axis of the ellipsoidal protein (Fig 6C). In contrast, CMMBP (Fig 6A) and Lmo0181 (Fig 6B) have a relatively hydrophilic area in the cleft, and the binding orientation of the glucotetrasaccharides (CMM or CNN) is parallel to the major axis of the protein molecule. A pocket accommodating a glucose unit is present in the C-domain side of CMMBP and Lmo0181, but no such pocket is present in TvuCMBP because of a wall formed by Glu170. CMMBP has a deeper pocket than that Lmo0181. When compared with the closed structure of Lmo0181 (Fig 5A), a closing movement of CMMBP on CMM binding may be triggered by a hinge-movement of the N-domain, which will form additional interactions with the Glc1 and Glc2 units in the cleft. This hypothesis is also supported by the more extensive interactions with the C-domain (mainly by stacking interaction with aromatic residues) compared with the N-domain (Fig 4C).

## Conclusion

In this study, we performed a functional and structural analysis of CMMBP (CmmC), a putative SBP involved in a bacterial ABC importer system. Functional analysis revealed that CMMBP specifically binds CMM with a high *K*_a_ value. Structural determination of CMMBP enabled structural comparison with other SBPs for carbohydrates. The structural features of CMMBP and Lmo0181 for binding cyclic glucotetrasaccharides (CMM or CNN) were basically similar, despite the very low amino acid sequence conservation (~20%, see Table 3). The CMMBP structure complexed with MM has an open conformation, suggesting that closure of the bilobed SBP structure requires binding of the canonical cyclic ligand (CMM). In the case of maltose transport system of *E*. *coli*, the substrate specificity is conveyed by the interface between maltose-binding protein in a closed conformation and the membrane transporter (MalFGK_2_) in a pre-translocation state [32], and the structure of maltose-binding protein complexed with a non-physiological ligand (β-cyclodextrin) was in a fully open conformation [33]. Therefore, the present study suggested that CMMBP plays a key role in the “three-stage” metabolic pathway of *A*. *globiformis* [14] because this protein is tuned for specific binding and intake of the cyclic compound, CMM. While CNN is formed from partial hydrolysate of starch (maltodextrin) by the actions of two enzymes of *L*. *monocytogenes* (α-6-glucosyltransferase and 3-α-isomaltosyltransferase) [25], the metabolic system of *A*. *globiformis* forms CMM directly from starch by the two-step actions of a single extracellular enzyme, 6-α-maltosyltransferase (CmmA) [10]. This type of metabolic pathway may be advantageous in the competition of carbon source acquisition by transiently changing the molecular form of a digestible glucan (starch) into an exclusive cyclic form, which is rarely assimilated by other microorganisms in the same biological niche.

## Supporting information

S1 Raw images (PDF)
