# Supplementary material for: Molecular analysis of cyclic α-maltosyl-(1→6)-maltose binding protein in the bacterial metabolic pathway
Source: PLoS One. 2020 Nov 19;15(11):e0241912. doi: 10.1371/journal.pone.0241912 (PMC7676653; doi:10.1371/journal.pone.0241912)
Supplement: S1 Raw images — (PDF) [file pone.0241912.s001.pdf]

Figure 2A original gel image

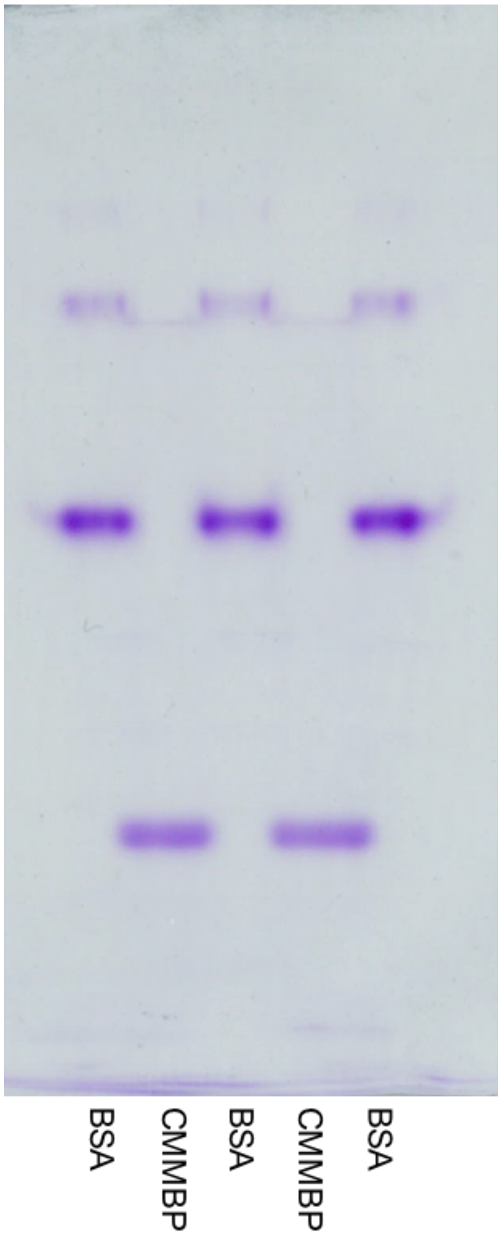

Native-PAGE without polysaccharides

Figure 2B original gel image

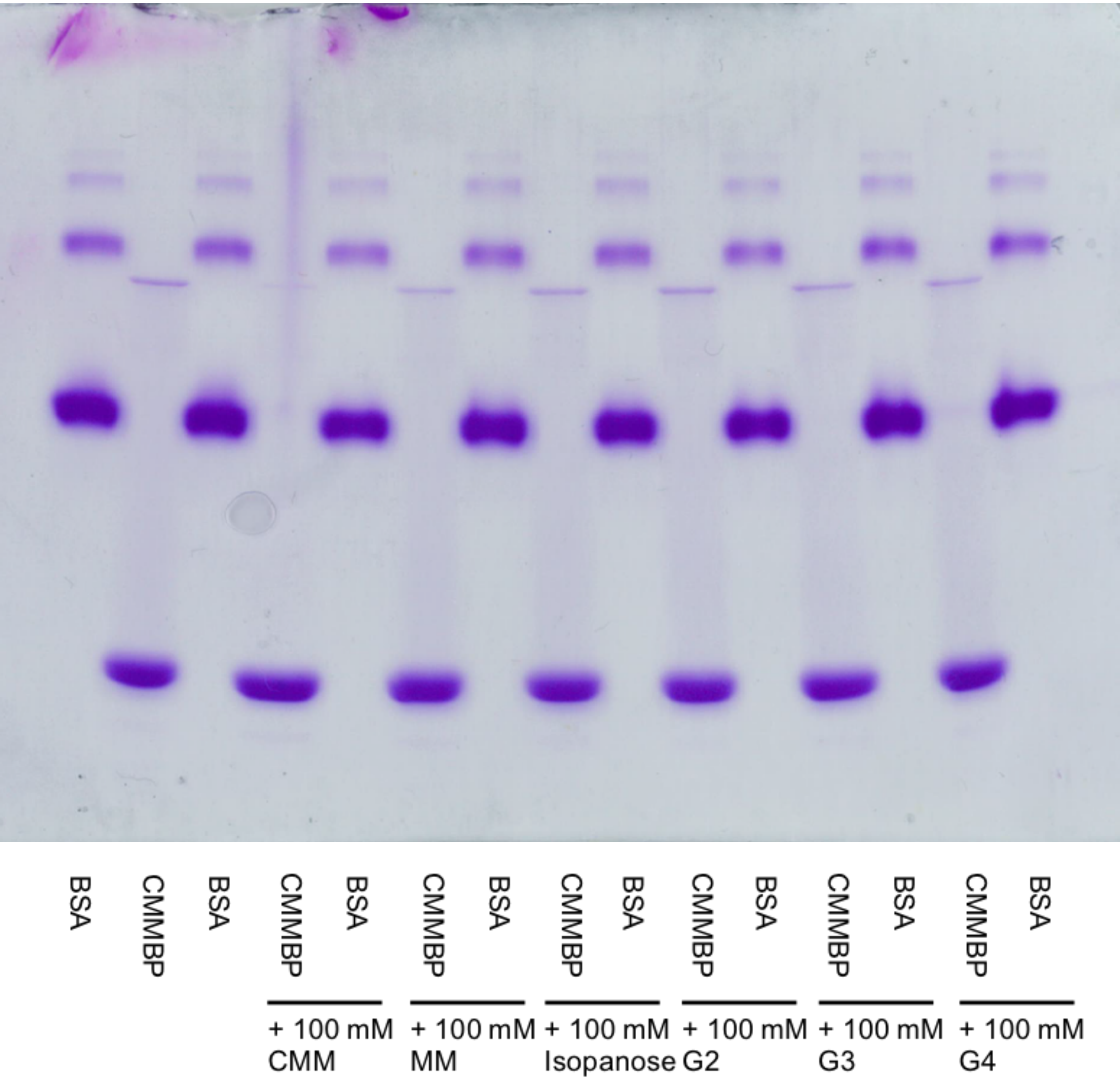

Native-PAGE in the presence of Pinedex #100

Figure 2C original gel image

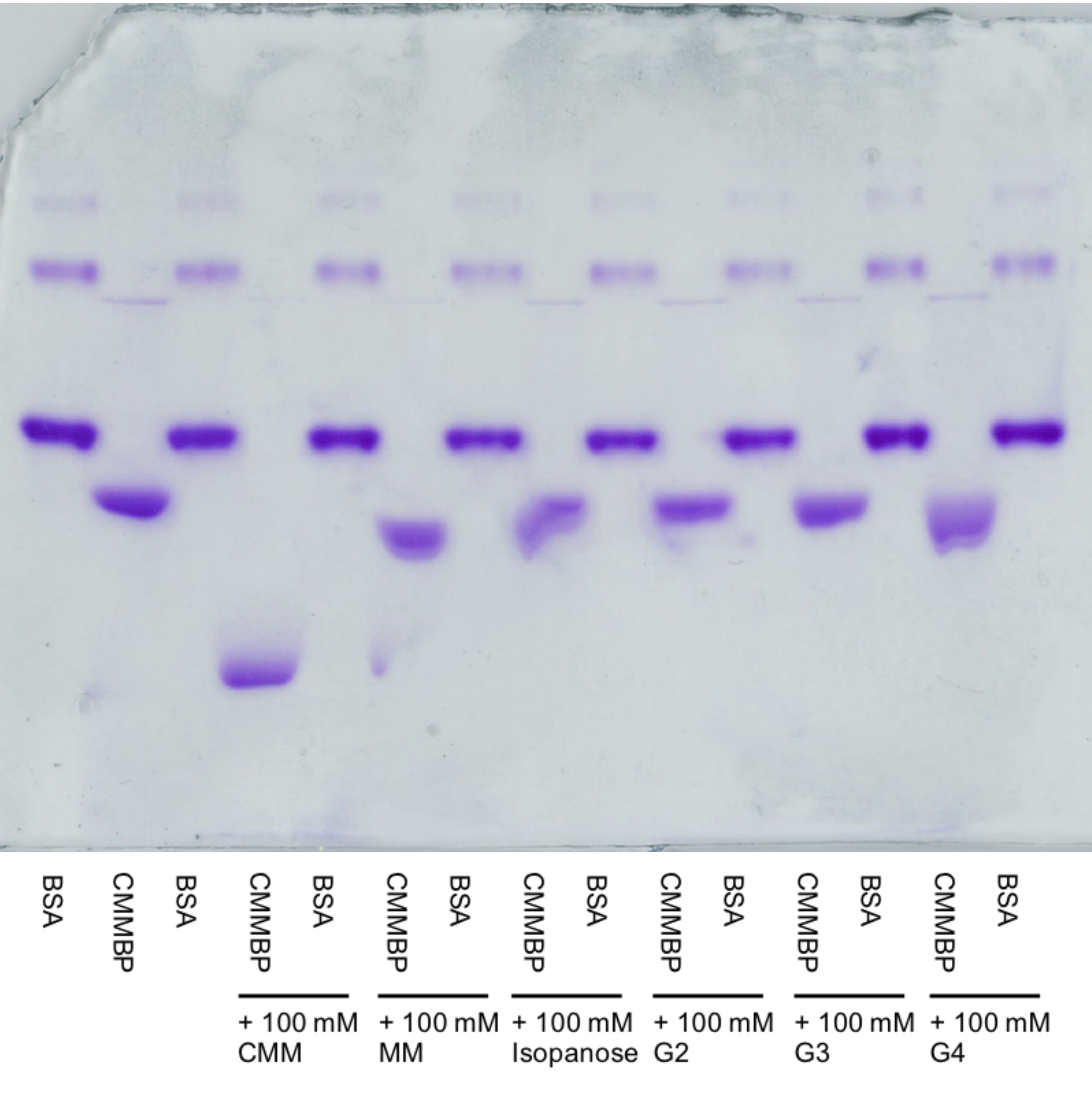

Native-PAGE in the presence of dextran (1)

Figure 2D original gel image

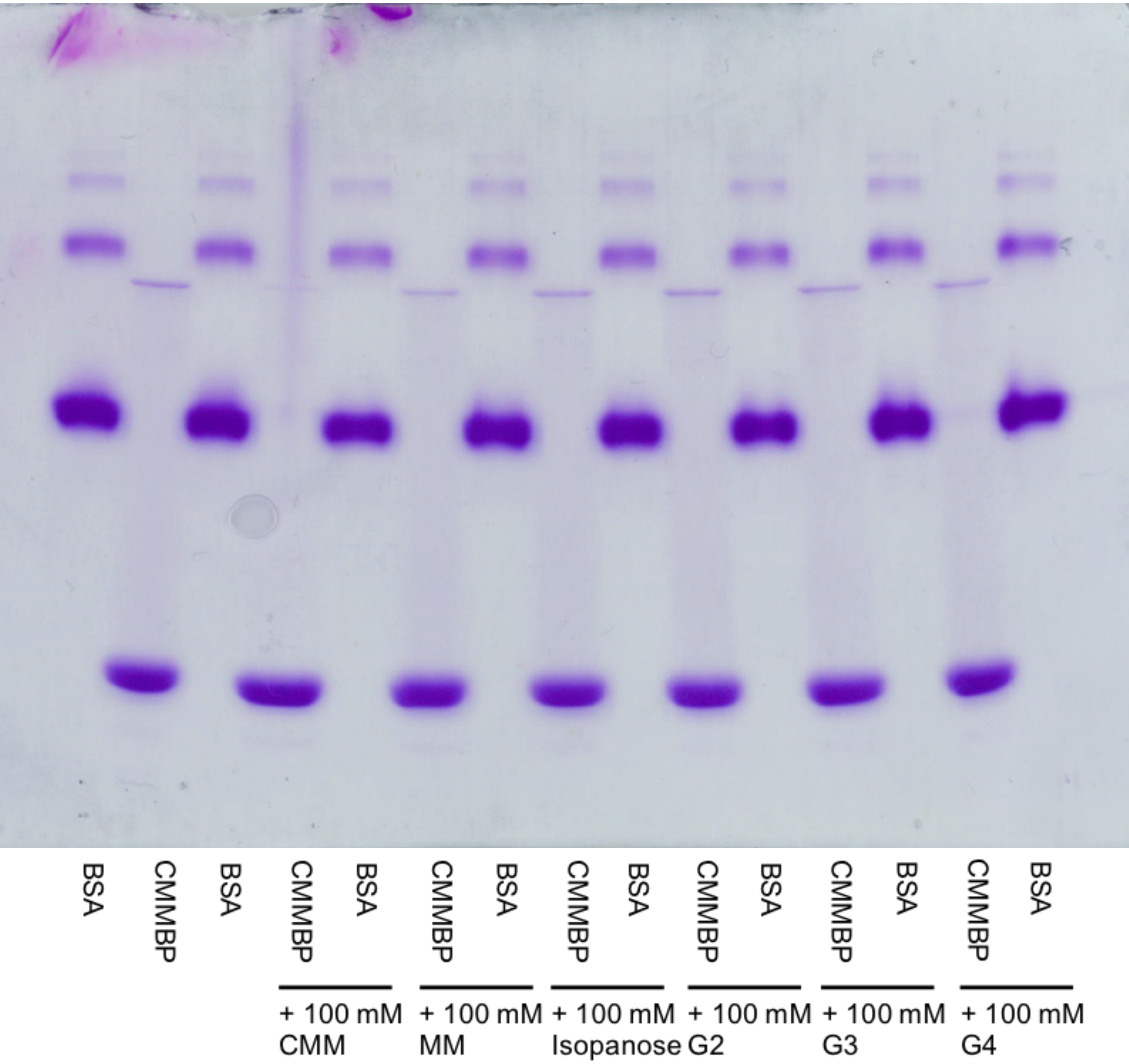

Native-PAGE in the presence of pullulan

Figure 2E original gel image

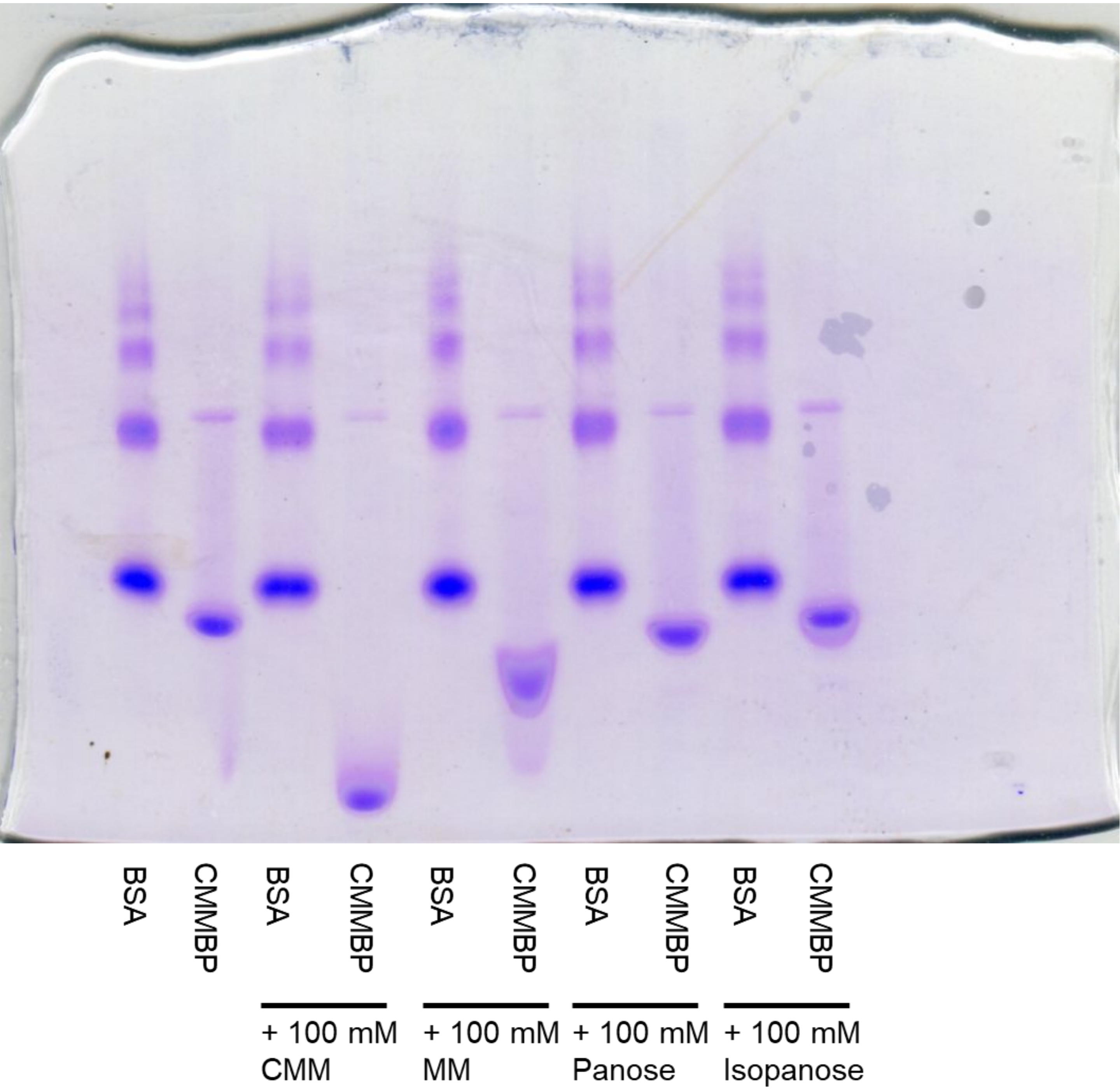

Native-PAGE in the presence of dextran (2)
